# Implementation research in LMICs—evolution through innovation

**DOI:** 10.1093/heapol/czaa118

**Published:** 2020-11-06

**Authors:** Kabir Sheikh, James Hargreaves, Mishal Khan, Sandra Mounier-Jack

**Affiliations:** 1 Alliance for Health Policy and Systems Research, World Health Organization, 20 Avenue Appia, 1217 Geneva, Switzerland; 2 London School of Hygiene and Tropical Medicine, Keppel Street, London WC1E 7HT, UK

Major global health gains can be achieved by strengthening the delivery of public health policies and programmes in low- and middle-income countries (LMICs). The population impact of evidence-based technologies and interventions such as drugs, vaccines and health know-how can only be maximized where programmes optimally identify and reach target populations and support them to take up and sustain their effective use. Examples include significant gaps in the coverage and quality of maternal health, newborn, immunization, non-communicable disease, primary care and adolescent sexual and reproductive health services—all issues tackled in this supplement. While structural change and increased funding are essential, much can be gained through ongoing improvements in programme delivery (Paina and Peters, 2012). Implementation gaps are also widely implicated in the failure of broader health policies and reforms in LMICs (Haines *et al.*, 2004), such as for decentralization, health care regulation and primary health care. This makes it important also to analyse the implementation of policies at all levels, including studying the negotiations and interactions of actors in social and political contexts, understanding gaps in the effectiveness of public policies and helping to resolve them.

Rigorous scientific studies of the implementation and effectiveness of public health programmes and policies delivered in real-life settings have long been acknowledged to be critical to accelerating impact and fostering innovation in this area (Fixsen *et al.*, 2005). This area of enquiry interchangeably referred to as implementation research (IR) and implementation science has captured widespread attention. Taking IR to scale is essential to support the delivery of public health programmes and broader reforms such as Universal Health Coverage. This supplement, ‘Innovations in Implementation Research in Low- and Middle-Income Countries’ showcases innovations in IR that are enhancing its value, shaping its development and fuelling the growth of the field. Specifically, we look to innovations that are occurring in LMIC contexts—where IR has the greatest potential to have impact. It does not seek to define IR, since we recognize that numerous authoritative texts have done so already. The supplement is a joint production of *Health Policy and Planning* and the Alliance for Health Policy and Systems Research.

Widely acknowledged as an eclectic area of enquiry not reliant on any one method or discipline, IR needs to adapt and innovate to meet the diverse demands upon it. Innovations are relevant at each stage—from developing more fit-for-purpose study designs to deploying multiple methods and disciplines to better effect, to innovations in fieldwork and analysis (Peters *et al.*, 2013a). The governance of IR, including the evolution of appropriate ethical standards, also represents a potential area of innovation (Gopichandran *et al.*, 2016). A further aspect of innovation has been in considering who participates in IR. The lack of alignment of existing research with the priorities and needs of their ultimate consumers (i.e. health system decision-makers and practitioners in LMICs)—is increasingly being recognized as widespread and counterproductive. To counter this misalignment, innovations such as ‘embedding’ IR into LMIC health systems, and participatory approaches involving implementers and practitioners, are gaining momentum (Ghaffar *et al.*, 2017).

The supplement consists of 12 articles that present innovations in the methods, approaches and governance of research on the implementation of public health policies and programmes in LMICs. Each of the papers illustrates the concept and usefulness of IR in different ways and this mix highlights its transdisciplinary character—defined by the real-world implementation challenges that it seeks to address and deploying a range of methodological inputs to analyse and tackle them. Two commentaries, one by country policymakers who have played roles in institutionalizing IR in their countries, and other by the leadership of WHO on the significance of IR in promoting cultures and practices of learning in health systems, complement the research articles. We hope that this supplement will help shape the trajectory of the development of the field and more importantly, help to chart the way forward for the further application of IR to maximize its impact on policies and programmes in the real world.

## Driven by the questions—the methodological spectrum in IR

The range of methods showcased in this collection of papers reflects the diversity of research questions that IR addresses, as well as its diverse disciplinary forebears. While IR may have found widest application in the study of healthcare services, its origins are diverse and rooted as deeply in the political science and public administration literature as in the health sciences (Hjern and Hull, 1982; Pressman and Wildavsky, 1984). It has also been informed historically by other subject areas—such as education and information technology—in which it has found useful application (Cooper and Zmud, 1990; Spillane *et al.*, 2002). As with the broader field of health policy and systems research, IR is question-driven rather than method-driven. Key questions range from the normative and evaluative to explanatory and even exploratory in nature (Sheikh *et al.*, 2011). This variety in intent as well as in methods is reflected in the 12 papers in the supplement.

The first two papers in the supplement are quality improvement studies linked to programme interventions. Manzi and colleagues use a realist perspective to evaluate and support a quality improvement initiative for a maternal health programme in Tanzania. The realist evaluation traced mechanisms triggered by the intervention, suggested course corrections and helped link broader programme theories with actionable implementation considerations. Lall and colleagues used mixed methods and conceptual frameworks for IR in a quality improvement intervention study for Non-communicable Disease care services in south India. Their qualitative findings showed how team dynamics were critical enablers of programme success. These papers highlight the importance of theories and frameworks in IR and their role in helping generate practicable knowledge to improve implementation processes.

The next two papers in the supplement share a focus on programme evaluation. Soi and colleagues describe their approach to mixing methods within Gavi full country evaluations and how these were adapted over time. As the evaluation questions become more focused on examining processes and diagnosing implementation breakdowns, their emphasis shifted towards improving programme learning. To support this, evaluation teams increased their level of embeddedness with immunization programmes. Peven and colleagues’ systematic review examines implementation strategies and implementation outcomes for essential newborn care in LMICs, highlighting challenges in generating learning from IR across settings due to the poor description of interventions and implementation outcomes.

The contributions by Suchman *et al.* and Parashar *et al.* are reminders of the political science lineage of IR, shedding light on contextual influences on the implementation and effectiveness of health programmes and policies. Suchman and colleagues examined the implementation of Kenyan UHC policies. Their innovation was to reanalyse data initially generated to improve programme implementation through a policy analysis lens. In doing so, they shed light on important gender issues and biases at policy level. Parashar and colleagues apply the trope of the implementation ‘black box’ and actor interface analysis to unpack the complex realities of implementation of a flagship maternal health policy in India. This analysis throws light on the influence of everyday power and politics on the policy process. The papers showcase IR in the sense of ‘sceptical enquiry into the structure and functions of policy processes’ (Hjern and Hull, 1982) with value in unearthing and tacking the hidden dynamics that often underlie surface phenomena.

## Boundary spanning—enhancing stakeholder engagement in IR

The latter six papers in the supplement are marked by innovations of a different type. These papers disrupt conventions around the actors and constituencies engaged in generating IR and its perceived target audiences.

Mbachu and colleagues document the experiences of academic researchers and non-academic implementers in Nigeria collaborating to design implementation strategies for adolescent sexual and reproductive health services. The authors highlight the benefits of research co-production in enabling the adoption of findings, but also caution readers on the complexities of collaboration across constituencies and the risks it poses to the fidelity of research outcomes. Varyallay and colleagues investigate the role of four key features of embedded IR in enhancing evidence to action processes, across three IR projects in Latin America and the Caribbean. The central involvement of policy/programme decision-makers and their collaborative partnerships with researchers—both central tenets of the embedded research approach—were noted by the authors to be critical in enabling the uptake of evidence.

The papers by Alonge *et al.* and Javadi *et al.* showcase the importance of learning collaborations with decision-makers across sectors—in these instances, education in West Asia and civil infrastructure in two African countries, respectively. With the Sustainable Development Goals highlighting how the determinants of health often lie outside of the health sector, there is a special role for IR in facilitating the delivery of cross-sectoral initiatives. Integrating shared learning goals into cross-sectoral collaborations proved to be important in these cases.

Ozano and colleagues report findings from participatory action research (PAR) on health systems strengthening for neglected tropical diseases in Nigeria and Liberia. Their PAR methodology provides useful lessons on integrating communities into learning processes at different levels of the health system. In the final paper of the supplement, Adam and colleagues highlight the importance of privileging community and user voices in complex health systems interventions, through human-centred design. Their study in Kenya tracked community health volunteers in the implementation of their action plans: the human-centred approach reimagines—and potentially helps re-engineer—services from the user perspective and in doing so helps builds trust between stakeholders.

## IR for real-world change—future directions

For an applied area such as IR, evolution takes place by documenting and recording innovations in research practice, and we hope that this supplement marks a step in that direction. In a seminal paper in 1980, Elmore expressed the concern that (most) IR was ‘long on description and short on prescription’ and that the advice from influential IR studies was often ‘desultory and strategically vague’ (Elmore, 1980). As we attempt to chart key principles that should drive the evolution of the field, an orientation towards change emerged as a key characteristic of the papers in this supplement—suggesting that Elmore’s concern might no longer apply, when it comes to IR on health policies and programmes. The papers demonstrate a strong focus on supporting change in policies and practices, often through embedded research practice. Advancing change through *learning*—whether in the short or long term—is a common thread. Valuing IR that promotes learning for change in policy and practice is of critical importance. What is clear from this collection is that such learning for change necessitates deep and wide engagement of diverse sets of stakeholders in the research process. It is crucial to align IR more closely with the demands of policymakers and programme managers within the health sector and in other related sectors, and with the needs of users and communities. This action-oriented, embedded perspective complements the emergence of in pragmatic, real-world community-randomized trials, also an area that has seen recent innovations (Geng *et al.*, 2019).

Most of these papers showcase the value of IR in improving the implementation of programmes and services by addressing specific, contextually determined problems and bottlenecks, and this remains a central contribution of IR to change. However, not all types of IR enable change through easily actionable solutions and prescriptions. As some other papers show (Adam *et al.*, Suchman *et al.*, Parashar *et al.*) underlying systemic issues—power, politics and the dominance of particular narratives—often shape and define visible implementation challenges, and the value of IR can lie not just in improving implementation but also in raising critical questions about the appropriateness of policies and programmes in the first place. In order to meet its full potential, IR must not be constrained by territorial boundaries, but continue to embrace diverse methodologies and approaches in addressing the full spectrum of questions about implementation.

## Disclaimer

KS is a staff member of the World Health Organization and is alone responsible for the views expressed in the article, which do not necessarily represent the views, decisions or policies of the World Health Organization.
